# Teeth Eruption Disorders: A Critical Review

**DOI:** 10.3390/children9060771

**Published:** 2022-05-24

**Authors:** Panagiotis Roulias, Nikolaos Kalantzis, Dafni Doukaki, Aspasia Pachiou, Konstantinos Karamesinis, George Damanakis, Sotiria Gizani, Apostolos I. Tsolakis

**Affiliations:** 1 Department of Orthodontics, School of Dentistry, National and Kapodistrian University of Athens, 11527 Athens, Greece; gdamanak@otenet.gr (G.D.); apostso@otenet.gr (A.I.T.); 2 Independent Researcher, 11527 Athens, Greece; nikoskalantzis21@gmail.com (N.K.); daphne.doukakis@gmail.com (D.D.); k.karamesinis@gmail.com (K.K.); 3 Department of Prosthodontics, School of Dentistry, National and Kapodistrian University of Athens, 11527 Athens, Greece; aspapachiou@gmail.com; 4 Department of Pediatric Dentistry, National and Kapodistrian University of Athens, 11527 Athens, Greece; stgizani@dent.uoa.gr; 5 Department of Orthodontics, Case Western Reserve University, Cleveland, OH 44106, USA

**Keywords:** primary failure of eruption, PFE, ankylosis, tooth eruption disorders

## Abstract

Dental eruption refers to the vertical displacement of a tooth from its initial non-functional towards its functional position. Tooth eruption disorders may be expressed in various clinical conditions, which may be grouped as “primary retention” and “secondary retention”. The purpose of this article is to review the literature and the clinical parameters of the various conditions related to tooth eruption disorders. Materials and Methods: The search strategy of this critical review included keywords in combination with MeSH terms in Medline, Scopus, and Cochrane Library until February 2022 and only in English. Results: “Primary Failure of Eruption” (PFE) occurs during the eruption process and includes clinical characteristics of both primary and secondary retention, which make diagnosis difficult. PFE is distinguished by Types I and II. In Type I, the defect in the eruption process occurs in all the relative teeth at the same time, whilst in Type II, the clinical expressions vary in multiple quadrants of the mouth, and the second molars erupt more. The variability of the PFE’s clinical spectrum seems to be connected to a genetic origin. The differential diagnosis among single ankylosis, secondary retention, and PFE is based on the occlusal relationship between the upper and the lower teeth distally, most commonly the first molar, which has not yet fully erupted. The treatment approach depends on many factors and combines surgical and orthodontic techniques.

## 1. Introduction

Dental eruption constitutes the physiologic process where a tooth is vertically displaced from its initial non-functional, developmental position towards its functional position, emerging through the bone of the alveolar process and the mouth epithelium, in order to occlude with its antagonist. This process is triggered by the formation of the periodontal ligament [1]. The tooth eruption path is dictated by the absorption forming the eruption path rather than the root formation rate. As soon as the eruption path is determined, the eruption procedure is initiated [2].

The physiological eruption procedure of one or more teeth is likely to be disrupted by local or genetic factors. Due to the slow rate of the tooth eruption and the fact that access to this ongoing process is deemed challenging, the exact procedure remains unclear [1]. Tooth eruption disorders can be manifested in several clinical conditions where the oral location, the number of the affected teeth, and the etiology of the disorders vary considerably. Eruption failure is often attributed to genetic factors. A variety of syndromes involving eruption failure have been mentioned, among which cleidocranial dysplasia, Gardner syndrome, osteoglophonic dwarfism, regional odontodysplasia, oculodental syndrome, Rutherfurd type, Nance–Horan syndrome, Cherubism, Albers-Schönberg osteopetrosis, McCune–Albright syndrome, hypodontia–dysplasia of nails syndrome, osteopetrosis, mucopolysaccharidosis, and GAPO syndrome [2]. In cleidocranial dysplasia patients, a combination of delayed tooth eruption and a high bone width of the upper and the lower arches was observed. In these patients, the paracrine signal for bone remodeling could account for the incomplete tooth eruption [3].

This article aims at reviewing the literature and presenting the causative factors and the clinical characteristics of the various conditions related to tooth eruption disorders.

## 2. Materials and Methods

This critical review was developed using a search strategy with the following keywords “tooth eruption disorders”, “PFE”, “primary failure of eruption”, “primary retention”, “secondary retention”, and “ankylosis”, which were combined with suitable Medical Subject Headings (MeSH terms) together with free text words in single or multiple conjunctions. An electronic search was conducted in Medline (PubMed), Scopus, and Cochrane Library databases supplemented with a hand search of studies published until February 2022.

Search results concerning animal studies, techniques, and writers’ personal opinions were excluded. Additionally, only articles in English language were selected without any time limit concerning the publication period. The initial data screening revealed 2948 results. In the first search phase, articles were collected when the title and the abstract matched the search goals. Each selected article was revised twice by two different reviewers (P.R., A.P.). Consequently, 34 articles were selected for inclusion in the present critical review.

## 3. Results

The finally selected articles numbered 34, and among them, 9 were clinical trials (comparative, prospective, or retrospective), and 3 were systematic reviews. Due to the lack of randomized controlled clinical trials available on this topic, some case reports of rather scarce entities were also included.

## 4. Eruption Failure

A non-normal tooth eruption path may occur from the presence of a given mechanical obstacle (with idiopathic or pathological origin) or as a result of the disruption of the tooth eruption mechanism itself [4]. The tooth eruption process involves a synchronized procedure where consecutive signaling events take place between the tooth pocket and the osteoblast and osteoclast cells of the alveolar process [5]. A disorder caused or not by a syndrome (of genetic or otherwise origin) might lead to the disruption of the aforementioned process, manifested either as a delayed eruption [6] or a complete eruption failure [7]. Eruption failure has been detected in either a single tooth or multiple teeth in both the primary and the permanent dentition and may also be partial or complete [8]. Apart from these factors, the eruption process of a permanent tooth could also be affected by genetic factors since numerous syndromes are found to give rise to similar clinical conditions where any genes responsible have already been discovered.

Tooth eruption failure may be referred to as either primary retention or as secondary retention [4]. Diagnosis of the initial eruption discontinuation concerns the local failure of a tooth to erupt without necessitating the coexistence of any other local or systematic factor. There has been an association between initial tooth eruption failure and idiopathic eruption failure [7]. Secondary retention refers to teeth that, although existing in the oral cavity, have manifested as an incomplete eruption [4,8].

## 5. Primary Retention

**Figure 1:**
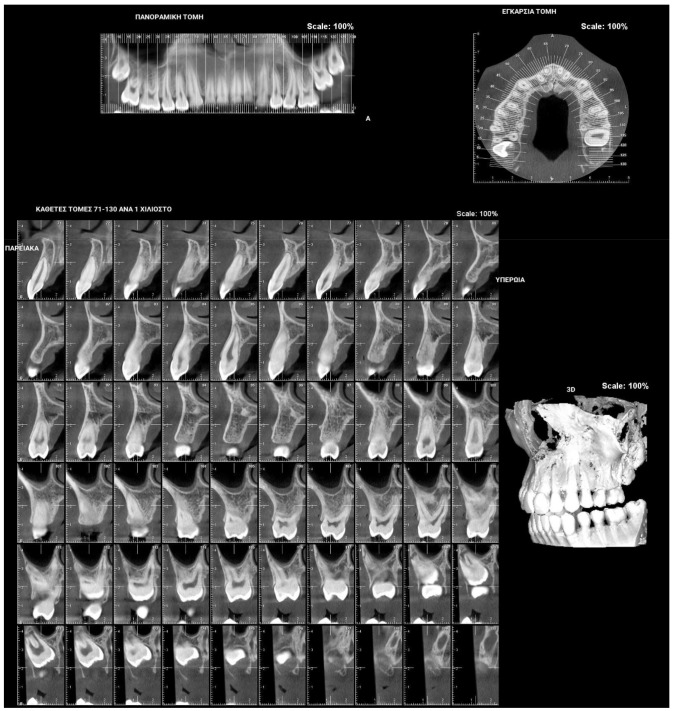
Patient presenting eruption failure of the upper left second molar due to primary retention.

**Figure 2:**
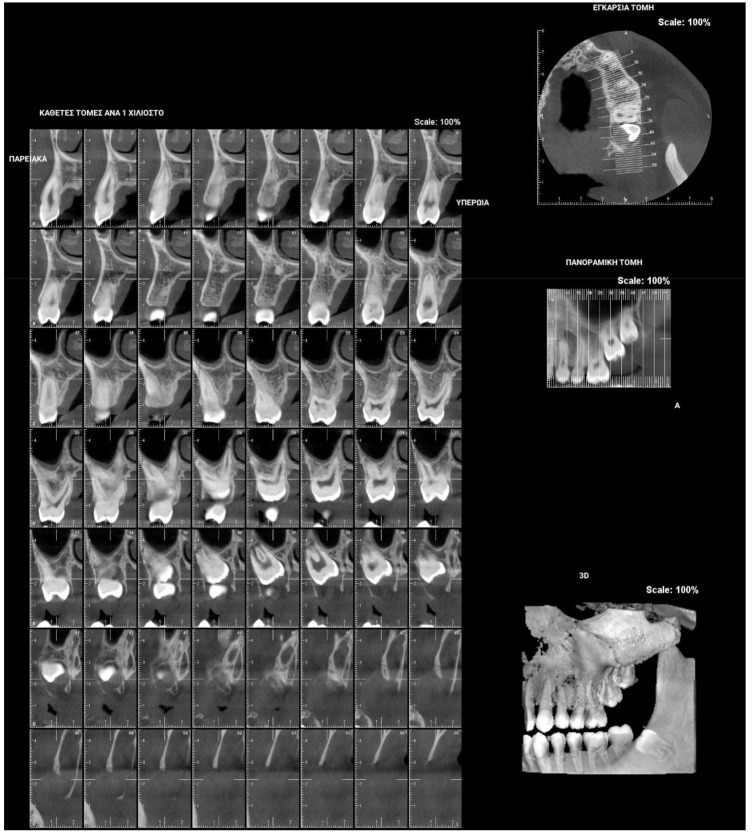
Patient presenting eruption failure of the upper left second molar due to primary retention.

The phenomenon where molars cease to erupt before they emerge, without a physical barrier in the eruption path or as a consequence of an atypical location, is defined as primary retention. The alveolar support of a primarily retained molar, which does not resorb occlusally, is considered a normal barrier to the eruption path. Primary retention is similar to “unerupted” and “embedded” teeth [9]. In the event that the eruption of a permanent tooth is at least 2 years delayed compared to what is normally expected, primary retention should be a clinical entity to take into account. A radiographical follow-up of a minimum of 6 months is suggested as an initial control to determine whether the tooth is showing any eruptive movement or not [10]. The suggested radiographic methods include a periapical or even panoramic X-ray. Primary retention is the possible result of a disturbance in the dental follicle that does not succeed in initiating the metabolic events responsible for bone resorption in the eruption traject [11] (Figure 1 and Figure 2).

## 6. Secondary Retention

Secondary retention is the phenomenon where the eruption process stalls after the tooth has emerged without the evidence of a physical barrier in the eruption path or as a result of an abnormal position [12,13]. The etiology of secondary retention still remains unclear. It takes place when the tooth is still submerged or during the eruption process and always involves the first 2 mm of the radicular neck or the molar furcation region, i.e., the area that separates the attachment cord from the follicular sac and the anatomic neck, a region approximately 2 mm wide. As soon as ankylosis between the tooth and osseous support has taken place, not only the eruption but also the growth of the alveolar process in the influenced area is inhibited. The adjacent teeth carry on erupting and, as a consequence, the affected tooth is infraoccluded. This might lead to malocclusion, particularly after the inclination of the adjacent teeth towards the space [4].

**Figure 3:**
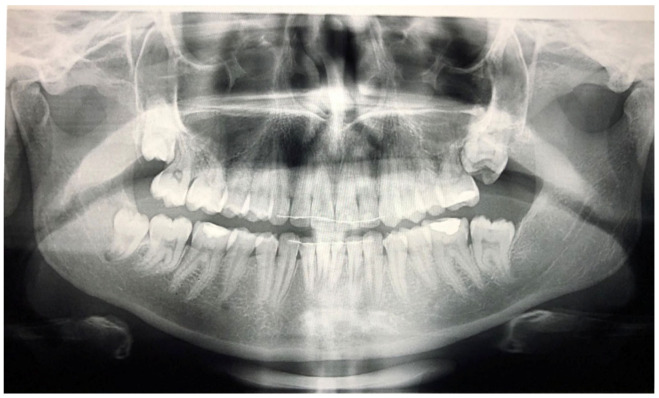
Patient after finishing the orthodontic treatment, presenting secondary retention due to fusion of the upper left second and third molars.

Clinically secondary retention is often assumed when a molar is in infraocclusion at a time period when the tooth should typically be in occlusion. The probability of ankylosis can be recognized via a percussion test and radiographic examination of the periodontal ligament obliteration. Nevertheless, such diagnostic means do not always prove reliable due to the fact that the ankylosed area in secondary retention is usually difficult to observe [12] (Figure 3).

## 7. Primary Failure of Eruption (PFE)

“Primary Failure of Eruption” (PFE) is the clinical condition that is associated with failure during the tooth eruption process. Primary Failure of Eruption may present a number of the following characteristics: (1) a higher frequency of appearance in the posterior teeth area rather than in the anterior region, (2) the teeth may begin to erupt towards occlusion and stop erupting halfway through the process, (3) both primary and permanent molars may exhibit such a clinical condition, (4) this clinical condition may be spotted on either one side or both sides in the mouth, (5) permanent teeth with such a condition are likely to be ankylosed, (6) the application of any orthodontic force may result in tooth ankylosis, and (7) this clinical condition may be present without any relevant family history [7].

**Figure 4:**
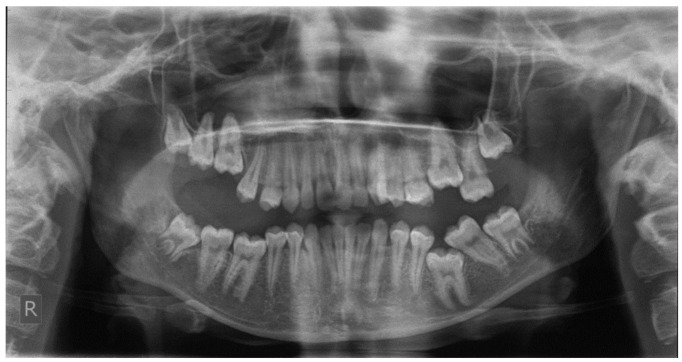
Patient’s orthopantomography presenting Primary Failure of Eruption in all four dental quadrants.

**Figure 5:**
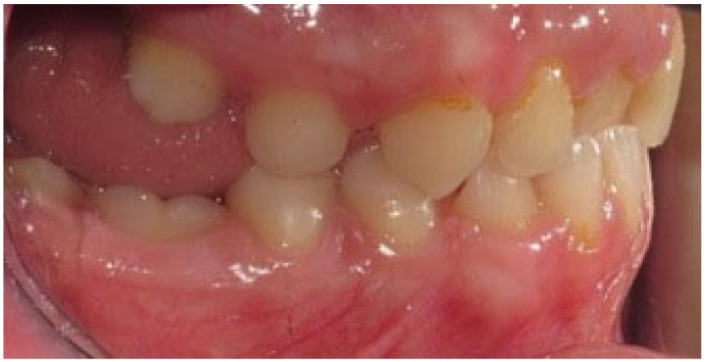
Right photo of the female patient with PFE in bite relationship.

**Figure 6:**
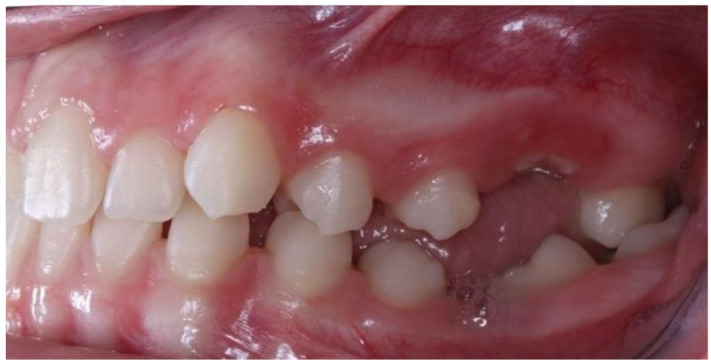
Left photo of the same female patient depicted in Figure 5.

PFE attributes combine elements that resemble both primary and secondary retention (Figure 4). The diagnosis of PFE is impeded to a great extent by the complexity of this clinical picture (Figure 5 and Figure 6). It appears that this clinical condition has two different mechanisms or two different aspects of the same mechanism since the tooth may erupt in its initial position and thereafter stop its further eruption (a clinical condition known as secondary retention) [7,8,14], or the tooth may not be able to erupt at all [11]. In this context, a definitive diagnosis of PFE cannot easily be decided, as it is possible that PFE presents two separate mechanisms [11] or two independent manifestations of the same mechanism. Only if we were to examine an environment where genetic, pathological, and environmental factors—all factors potentially responsible for the discontinuation of the tooth’s eruption—are absent would a PFE diagnosis through a retrospective examination be possible.

Open bite is present at the right posterior segment due to the impaction of the upper and lower left first molars.

The incidence of PFE is 1/2000 [15], while the average age of patients diagnosed with PFE is 13.65 years old. More often than not, the teeth with the higher PFE rate (excluding the third molars) are the first and the second molars in all four quadrants of the mouth [16]. Proffit and Vidd (1981) came to the conclusion that PFE may be manifested in all teeth but with an increased incidence in the posterior teeth, whereas Palma et al. (2003) narrowed it down to the first and second molars [17].

## 8. Genetic Background of PFE

Research findings of Proffit and Vid (1981) indicate that there is no correlation between PFE and a genetic background or relative family history; however, there has been a significant discrepancy between this conclusion and the findings of similar studies that suggest otherwise [18]. According to the latter, the presence of PFE in family history and the relation to a dental abnormality with a genetic basis could suggest that PFE has a strong genetic background. If the responsible gene is present, proteins that are active in the embryonic period and in the period following the child’s birth could lead to PFE [16]. A study investigating the characteristics of incisors for which the eruption process has stopped concluded that the presence of non-erupted incisors is more prevalent in boys than in girls with a 2.7:1 ratio. In addition, the same study suggested that the discontinuation of the incisor’s eruption occurs in parallel with other hereditary dental anomalies, such as enamel hypoplasia, supernumerary teeth, and dislocated teeth [19]. The PTH1R gene seems to be responsible for eruption failures that are not associated with any physical obstacles [20].

## 9. Clinical Characteristics of PFE

The variability in the clinical figure of PFE appears to have a genetic background and depends on the moment the defect of the tooth eruption occurs (which is also governed by genetic factors).

PFE is described as occurring in two distinct types: Type I is observed when a progressive open bite from the anterior towards the posterior area of the dental arches is established. It seems that in this case, the defect in the eruption process occurs in all the relative teeth simultaneously. In Type II, a progressive open bite from the anterior towards the posterior part is also observed, but the clinical expressions vary in multiple quadrants of the mouth, and the second molars erupt further but not sufficiently. It seems that in this case, a sequence of molecular events takes place, thus affecting the alveolar process in the posterior region more than in the anterior segment in terms of time and location [6]. Whenever Type I and II PFE are simultaneously present, a Type III PFE can be described [2].

## 10. Differential Diagnosis of Teeth Eruption Disorders

The common element for any differential diagnosis among single ankylosis, secondary retention, and PFE is the occlusal relationship (if it is normal or not) between the upper and the lower teeth posteriorly, with the first molar being the most likely not to have fully erupted. PFE is more likely to be present if a family history in conjunction with the mutated PTH1R gene are also present. In cases where PFE is present, the affected teeth are expected to not respond to any orthodontic treatment. If a patient is believed to have PFE, a genetic test for a mutation in the PTH1R gene should be recommended before any orthodontic therapy so as to prevent ankylosis. Therefore, a definite diagnosis based on the clinical description of PFE may not be easily reached. From the authors’ point of view, careful consideration should be given to the existence of possible clinical manifestations, and an observation period would be advisable. If the presence of such symptoms persists, a further genetic background search would be advisable.

The therapeutic approach is determined by the patient’s age and the clinical status, and, as such, it needs to be evaluated individually [2]. Considering that ankylosis concerns a single tooth, therapy could comprise the extraction of the ankylosed tooth and the subsequent orthodontic treatment for the space closure management or a prosthetic rehabilitation [21]. Alternatively, a local osteotomy, as well as an orthodontic traction of the whole piece, could be performed since a harmonic occlusion of the tooth may be achieved. In the event of the tooth’s partial eruption, the vertical dimension of the tooth in question could be regained by having its crown restored. When more than one tooth is affected, the treatment becomes increasingly challenging, and partial osteotomy is the preferred treatment approach [7].

## 11. Treatment Approach of Primary and Secondary Retention

The kind of treatment is chosen based on the following criteria: the presence of a pathologic process, the age of the patient, the position of the molar in the jaw relative to other structures, and the willingness of the patient to undergo treatment. In most cases, early surgical exposure of the crown is the best initial treatment [22,23,24].

The suggested therapeutic plan involves extraction of the third molar at the age between 11 and 14 years old [25], along with a meticulous check of the eruption of the second molar. For an impacted second molar that affects the first molar adversely, surgical or orthodontic repositioning with or without extracting the neighboring third molar is often suggested. Some clinicians recommend removing the second molar in order to allow for the eruption of the third molar in the expected location of the second molar or transplantation of the third molar into the socket of the removed second molar [26]. When surgery is carried out before the finalization of root formation, spontaneous eruption and prolongation of root development can then be anticipated [27]. Luxation of the molar after exposure has been additionally recommended to support eruption [27,28], yet it is perhaps needless as exposure solely is generally adequate to implement spontaneous eruption [13].

The therapeutic plan for secondary retention (idiopathic ankylosis) is influenced by the patient’s age as well as the degree of infraocclusion and malocclusion. Spontaneous eruption of a secondarily retained molar may happen but is extremely rare [13].

Orthodontic reposition of the affected molar is not feasible due to an atypical periodontal ligament [7]. If secondary retention occurs before the growth spurt, immediate extraction of the affected molar and then orthodontic alignment of the adjacent teeth is the treatment of choice. When secondary retention occurs during the growth spurt, the molar should be monitored at a 6-month follow-up. During or after the growth spurt, no active treatment other than a restorative build-up is recommended in cases where the adjacent teeth do not show inclination and the extent of infraocclusion is limited and stable [29]. In all other cases, the involved molar should be extracted; the edentulous diastema that occurs can close spontaneously or may need to be closed orthodontically or prosthetically. Alternatively, a developing third molar could be transplanted into this space [30].

## 12. Treatment Approach of Primary Failure of Eruption

Treatment of PFE is hindered by the fact that its diagnosis is difficult. Indeed, its diagnosis can only be made possible after the potential etiologic factors have been ruled out and the attempt to apply orthodontic forces on a tooth has been unsuccessful, hence resulting in ankylosis [7]. In this case, the PFE diagnosis is corroborated by X-rays in which the periodontal ligament is absent; a lack of normal tooth mobility; and a solid, sharp sound of the tooth [22]. However, the mere absence of the periodontal ligament could wrongly lead to the diagnosis of ankylosis; in such cases, the detection of the mutated gene PTH1R may result in the right final diagnosis [2].

Figure 5 and Figure 6 present a PFE case of a 17-year-old female patient referred to the Orthodontic Clinic of the Dental School of the National and Kapodistrian University of Athens, Greece. Clinical examination showed a posterior open bite on the right segment, as well as infraoccluded left upper and lower first molars. The patient’s orthopantomography shows no visible PDL of the right upper and lower first molars or the teeth distally related to them.

The treatment approach to PFE conditions may include a wide spectrum of choices. Whenever such a diagnosis is made, careful consideration should be taken whether it should be treated or the clinician should rather observe the infraocclusion [31]. If the treatment is manageable at an age that the growth has already been completed, a prosthesis could be a suitable therapeutic option. An onlay or a build-up of the crown of the affected tooth should be considered. Alternatively, a removable partial overdenture that overlaps the infraoccluded tooth could be a treatment of choice [32,33,34].

Moreover, the tooth presenting PFE could be extracted and replaced by a prosthesis. Finally, a surgical approach could be a suitable treatment in such cases, with segmental osteotomy and repositioning of the infraoccluded area. Nevertheless, osteotomy in these cases does not present high chances of success [32,33,34].

## 13. Conclusions

The differential diagnosis of single ankylosis, secondary retention, and PFE is mostcommonly based on the occlusal relationship between the upper and the lower teeth distally from the first molar, which has not yet fully erupted. Clinical observation and radiographic follow-up constitute the initial diagnostic approach to such clinical manifestations. Diagnosis should be given after careful evaluation of any etiologic factors. Both orthodontic and surgical procedures may contribute to the therapeutic approach. Further research with clinical trials is required regarding possible treatment modalities of poorly defined eruption conditions. Additionally, more studies with a larger number of patients are essential to further investigate the suspected genetic involvement using multivariate analysis.

## Data Availability

Not applicable.
